# mRNA Expression and DNA Methylation Analysis of Serotonin Receptor 2A (*HTR2A*) in the Human Schizophrenic Brain

**DOI:** 10.3390/genes8010014

**Published:** 2017-01-04

**Authors:** Sern-Yih Cheah, Bruce R. Lawford, Ross McD. Young, Charles P. Morris, Joanne Voisey

**Affiliations:** 1 School of Biomedical Sciences, Institute of Health and Biomedical Innovation, Queensland University of Technology, 60 Musk Ave., Kelvin Grove, Queensland 4059, Australia; sern.cheah@hdr.qut.edu.au (S.-Y.C.); lawford.b@gmail.com (B.R.L.); p.morris@qut.edu.au (C.P.M.); 2 Discipline of Psychiatry, Royal Brisbane and Women’s Hospital, Herston, Queensland 4006, Australia; 3 Faculty of Health, Institute of Health and Biomedical Innovation, Queensland University of Technology, 60 Musk Ave., Kelvin Grove, Queensland 4059, Australia; rm.young@qut.edu.au

**Keywords:** *HTR2A*, schizophrenia association, mRNA expression, DNA methylation, rs6314, rs6313, epigenetics

## Abstract

Serotonin receptor 2A (*HTR2A*) is an important signalling factor implicated in cognitive functions and known to be associated with schizophrenia. The biological significance of *HTR2A* in schizophrenia remains unclear as molecular analyses including genetic association, mRNA expression and methylation studies have reported inconsistent results. In this study, we examine *HTR2A* expression and methylation and the interaction with *HTR2A* polymorphisms to identify their biological significance in schizophrenia. Subjects included 25 schizophrenia and 25 control post-mortem brain samples. Genotype and mRNA data was generated by transcriptome sequencing. DNA methylation profiles were generated for CpG sites within promoter-exon I region. Expression, genotype and methylation data were examined for association with schizophrenia. *HTR2A* mRNA levels were reduced by 14% (*p* = 0.006) in schizophrenia compared to controls. Three CpG sites were hypermethylated in schizophrenia (cg5 *p* = 0.028, cg7 *p* = 0.021, cg10 *p* = 0.017) and *HTR2A* polymorphisms rs6314 (*p* = 0.008) and rs6313 (*p* = 0.026) showed genetic association with schizophrenia. Differential DNA methylation was associated with rs6314 and rs6313. There was a strong correlation between *HTR2A* DNA methylation and mRNA expression. The results were nominally significant but did not survive the rigorous Benjamini-Hochberg correction for multiple testing. Differential *HTR2A* expression in schizophrenia in our study may be the result of the combined effect of multiple differentially methylated CpG sites. Epigenetic *HTR2A* regulation may alter brain function, which contributes to the development of schizophrenia.

## 1. Introduction

The serotonin receptor 2A (*HTR2A*) is an important signalling factor implicated in high-order cognition [1,2]. It is found expressed abundantly in the glutamatergic interneurons and gamma-aminobutyric acid (GABA)-ergic neurons in the prefrontal cortex and hippocampal regions and both neurotransmission systems are known to be involved in the pathogenesis of schizophrenia [3,4,5,6]. The serotonin receptor 2A gene (*HTR2A*) was shown to influence prefrontal cognition via binding of receptor agonists including quipazine and 3-trifluoromethylphenylpiperazine [7]. Deficits in prefrontal cognitive function (such as executive roles and working memory) are a core feature of schizophrenia [8]. In a later review, it was suggested that HTR2A antagonists were used as treatments aimed to improve cognitive function, although the efficacy of HTR2A antagonists has yet to be unequivocally established [9]. Overall, this evidence strongly suggests a role for *HTR2A* in the pathogenesis of schizophrenia.

Based on pharmacological and expression studies, results suggest that the downregulated *HTR2A* mRNA expression and reduction of receptor density or activity are associated with schizophrenia [7,10,11,12]. According to a review by Selvaraj et al., nine frontal cortex studies found 54 patients with decreased HTR2A receptor activity in schizophrenia [13]. However, one cohort of antipsychotic-free patients revealed upregulation of HTR2A receptor density in the prefrontal regions [14,15]. Maple et al. suggested that stress-induced expression of *HTR2A* as an adaptive function and is disrupted in schizophrenia patients. Although the induced expression of *HTR2A* may appear beneficial, along with the majority of the evidence pointing to the increased expression of *HTR2A* in healthy individuals, it is rather paradoxical that antipsychotics block the action of HTR2A [16]. This paradox is further highlighted by the fact that hallucinogens (such as lysergic acid diethylamide) are HTR2A agonists, while atypical antipsychotics are antagonists [16]. While clozapine can be considered an antagonist, it eventually triggers downstream activation of Akt, similar to the effect of serotonin agonists via different mechanisms [17]. Although such inconsistency remains to be clarified, these studies suggest an association between the dysregulation of *HTR2A* mRNA expression and schizophrenia.

The *HTR2A* polymorphism, rs6314 is a non-synonymous DNA variant located in exon 3 that results in a His452Tyr substitution. Studies suggest that rs6314 may have an effect on calcium signalling and mobilisation and altered activation of phospholipases C and D, possibly resulting in reduced receptor activity [18,19], but it is not clear how this impacts on neurotransmission or susceptibility to schizophrenia. Serretti et al. reviewed a number of association studies between rs6314 and schizophrenia and found inconsistent results [20]. Decreased *HTR2A* expression was associated with rs6314 in one study [21] and the polymorphism was also associated with a number of endophenotypes including hippocampal volume and activity [22,23], memory [23,24], and clozapine treatment response in patients with schizophrenia [25].

A synonymous *HTR2A* polymorphism, rs6313 (T102C) is a well-studied variant that was found to be in linkage disequilibrium (LD) with another functional polymorphism (rs6311) known to alter *HTR2A* promoter activity [26]. A number of studies have either found [12,27,28] or failed to find an association [10,29] between rs6313 and schizophrenia. Poorer cognitive performance was found to be associated with the T-allele of rs6313 [30]. However, another study found that poorer visual sustained attention was associated with the C-allele [31]. These different findings may be due to significant ethnic differences between the two studies. Binding activity or receptor density of HTR2A in the brain [32,33] and differential expression in the temporal cortex [12] are associated with the rs6313 polymorphism. Although the biological mechanism of rs6313 and rs6314 remains unclear, the findings suggest that both polymorphisms are good candidates for schizophrenia risk.

DNA methylation is thought to be an important epigenetic mechanism in schizophrenia [34] as environmental influences on *HTR2A* DNA methylation are associated with infant neurobehavioural outcomes [35]. For *HTR2A,* the majority of the DNA methylation activity occurs within the promoter and exon I, but there is little DNA methylation activity in other regions of the gene. *HTR2A* CpG sites were either hypermethylated (near rs6311, at position −1438 of the promoter region) or hypomethylated (near rs6313, at position 102 of exon I) in the prefrontal cortex of patients with schizophrenia, potentially resulting in downregulation of *HTR2A* expression in patients with schizophrenia [10]. Another study also reported similar results in the saliva of patients with schizophrenia [36]. Increased DNA methylation at rs6313 in peripheral leukocytes was reported in major psychosis patients with suicidal tendency [37], suggesting the potential involvement of *HTR2A* promoter hypermethylation in psychosis. Based on the literature, there is strong evidence of schizophrenia-specific DNA methylation changes in *HTR2A* that influence mRNA expression.

While it is clear that DNA methylation alters *HTR2A* mRNA expression [10,38,39,40] (apart from other known factors such as stress, medication and substance use, nutrition history, comorbidities and other underlying biological factors), *HTR2A* polymorphisms have also been reported to influence mRNA expression, possibly by altering recognition of CpG sites. One study [21] found that *HTR2A* rs6314 was associated with mRNA expression changes, although the effects of promoter DNA methylation were not taken into account. In this study, we investigated the role of *HTR2A* in schizophrenia by examining rs6314 and rs6313 genotype, DNA methylation, and the expression of *HTR2A* mRNA in prefrontal cortex samples of schizophrenia patients.

## 2. Materials and Methods

### 2.1. Samples

The brain tissue was provided by the Human Brain and Spinal Fluid Resource Centre, Los Angeles, California (courtesy of James Riehl). The brain tissues were collected from 25 schizophrenia patients and 25 healthy controls. Each sample consisted of a 7-mm coronal section that had been quick-frozen, followed by dissection of the frontal cortex (0.4–1.0 g) from the frozen sections. A summary of sample age, post-mortem interval (PMI) and gender are shown in Table 1, with complete sample information detailed in Supplementary Table S1. PMI represents the time interval from the death of a patient to the quick-freezing of the brain section. All but two of the patients with schizophrenia were using medication at their time of death. Five patients with schizophrenia were identified as suicide victims.

Extraction of RNA and DNA from post-mortem frontal cortex brain tissues was performed at the UCLA Clinical Microarray Core Laboratory (Los Angeles, CA, USA) using the Roche MagNa Pure Compact. There were five samples with missing mRNA data due to quality control failure, data generation failure and insufficient/no RNA content. Two samples had missing DNA methylation data as they were excluded due to protocol constraints (we could only run 48 samples due to the bead chip plate format).

Ethics approval for the project was obtained from the Human Research Ethics Committee of the Queensland University of Technology.

### 2.2. mRNA Data Generation

Sequencing of total RNA for each sample was performed by the Australian Genome Research Facility (AGRF). Quality of extracted RNA was assessed by electrophoresis using Agilent Bioanalyzer RNA 6000 Nano assay (Santa Clara, CA, USA). The percentage of RNA fragments >200 nucleotides was assessed to determine the appropriate input. Samples with <30% of RNA >200 nucleotides were excluded. A total of 46 samples passed the initial quality control (QC). Following sample QC, samples were processed and used to generate *HTR2A* mRNA sequence data with the TruSeq RNA Access Sample Preparation Kit as per the manufacturer’s instructions (Illumina, San Diego, CA, USA). Briefly, total mRNA was firstly fragmented, followed by first and subsequently second strand cDNA synthesis. The blunt-ended cDNA were 3′-adenylated and ligated with adapters that hybridise to the flow cell. The adapter-added cDNA were subsequently amplified creating a cDNA library. One sample failed to generate enough library to proceed to capture hybridisation and was excluded from the final analysis. A total of 45 samples were further investigated.

Captured libraries were pooled for sequencing. Each pool of libraries was clustered on the Illumina cBot system (Illumina, San Diego, CA, USA) using HiSeq PE Cluster Kit v4 reagents (Illumina, San Diego, CA, USA) followed by sequencing on the Illumina HiSeq 2500 system with HiSeq SBS Kit v4 reagents with 159 cycles (75 base pair paired end reads). Illumina RTA 1.18.61 (Illumina, San Diego, CA, USA) was used for base calling, quality scoring. Bcl2fastq pipeline 1.8.4 (Illumina, San Diego, CA, USA) was used for de-multiplexing and FASTQ file generation. 

### 2.3. mRNA Data Transformation

The total generated sequence reads in FASTQ files were mapped to the human genome (GRCh38/hg38) using Tophat 2.0.13 [41] to identify sequence reads that align/correspond to the *HTR2A* gene. Mapped RNA sequence reads aligned to *HTR2A* were subsequently counted using the tool feature Counts from the SubRead package 1.4.6p5 [42] to assign raw non-normalised read counts to genes (specifically *HTR2A*). Non-normalised read counts were used for the edgeR package 3.12.0 [43] to perform quality control and normalisation using the TMM (trimmed mean of M values) method [44]. Normalised read counts (expressed as reads per kilobase transcript per million mapped read counts or RPKM) were transformed to log_10_RPKM using SPSS (Statistical Package for the Social Sciences) 22 (SPSS, Chicargo, IL, USA).

### 2.4. Genotyping

**Figure 1:**
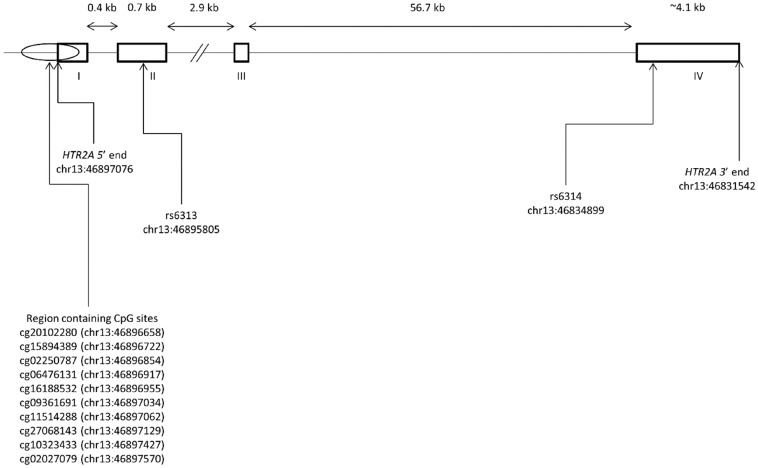
*HTR2A* gene structure showing exons, loci of rs6313 and rs6314, and all 10 selected CpG sites.

Total RNA sequence reads were aligned to GRCh38/hg38 using BWA (Burrows-Wheeler Alignment) for Illumina version 1.2.3 (Digital Equipment Corporation, Palo Alto, CA, USA). Genotypes of rs6314 and rs6313 (loci shown in Figure 1) were determined by visualising the aligned sequence reads using IGV (Integrative Genomics Viewer) version 2.3.52 (Broad Instituite, Cambridge, MA, USA). Polymorphism quality was further screened for its span within reads with spans at or near ends of reads excluded due to high sequencing error rates at ends of reads [45].

### 2.5. CpG Site Methylation Data Generation and Transformation

Genome-wide DNA methylation was analysed using the Illumina HumanMethylation450 array that assays more than 485,000 CpG sites across the genome exactly as previously described [34,46]. *HTR2A* methylation profiles were generated by selecting CpG sites within regions with active DNA methylation (±500 bp of transcription start site) including the promoter and exon I of *HTR2A*. These sites were selected for their loci within DNA regions with rich presence of regulatory signals/elements based on the UCSC genome browser. The sites were identified as cg20102280, cg15894389, cg02250787, cg06476131, cg16188532, cg09361691, cg11514288, cg27068143, cg10323433 and cg02027079 as detailed in Table 2 (loci shown in Figure 1). The methylation status for each site was recorded as a β-value that ranged between 0 and 1, where values close to 1 represent high levels of methylation and where values close to 0 represent low levels of methylation. β-values were transformed to logit values or log_10_(β/1−β) values using SPSS 22.

### 2.6. Data Analysis

Using the log_10_RPKM values to represent *HTR2A* mRNA expression levels and log_10_(β/1−β) values to represent CpG site DNA methylation levels, analysis of variance (ANOVA) adjusted for age and PMI (placing age and PMI variables as covariates) was performed with SPSS 22 to compare *HTR2A* mRNA expression differences and DNA methylation differences for the CpG sites between schizophrenia and control subjects. ANOVA was also used to examine the effect of rs6314 and rs6313 genotypes on *HTR2A* mRNA expression and DNA methylation of the CpG sites. Linear regression analysis adjusted for age and PMI was performed using SPSS 22 to correlate *HTR2A* expression levels and *HTR2A* methylation levels. *HTR2A* mRNA expression and DNA methylation for the CpG sites were firstly correlated in total samples, followed by correlation in schizophrenia-only and control-only subjects.

## 3. Results

### 3.1. Schizophrenia and HTR2A mRNA Expression

**Figure 2:**
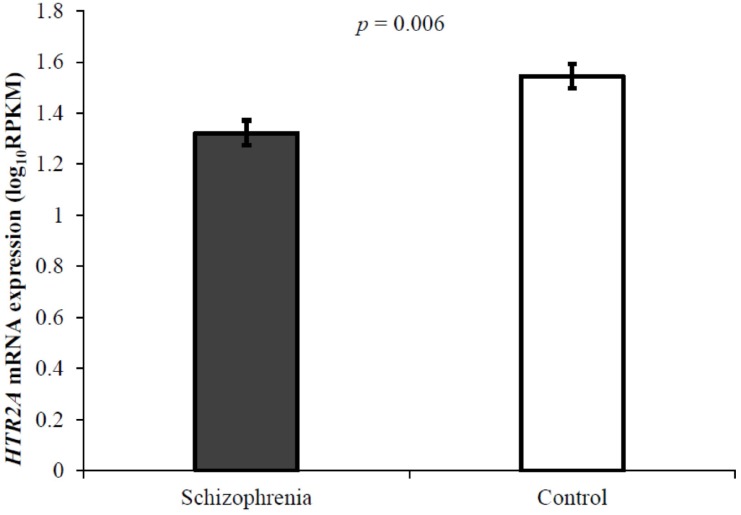
*HTR2A* mRNA expression in patients with schizophrenia compared to controls. ANOVA showing mean expression level and standard error in patients with schizophrenia.

The log_10_RPKM values were compared between schizophrenia and control subjects using ANOVA. There was a 14% reduction in *HTR2A* mRNA expression in patients with schizophrenia compared to controls (*F*-value of 8.564 and *p*-value of 0.006; Figure 2).

### 3.2. Schizophrenia and DNA Methylation

**Figure 3:**
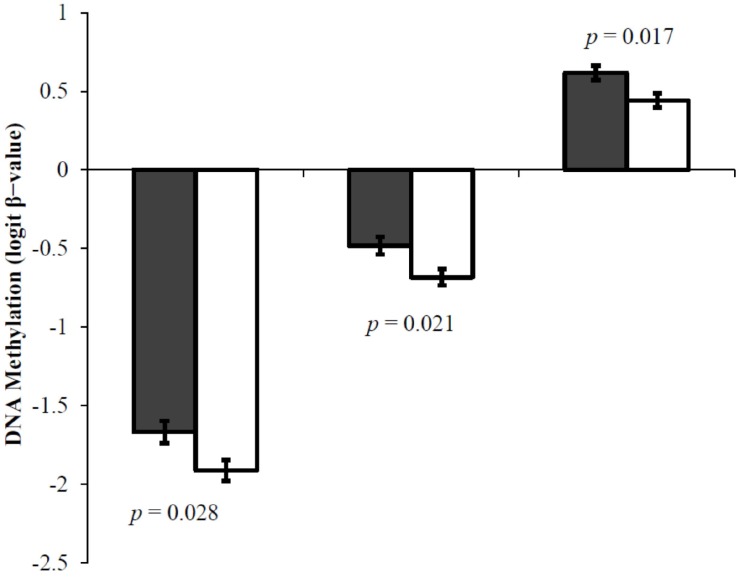
*HTR2A* DNA methylation in patients with schizophrenia compared to controls for three CpG sites, cg5, cg7 and cg10. ANOVA showing mean DNA methylation level and standard error in patients with schizophrenia (solid dark-grey bar) and controls (solid white bar).

Methylation levels (logit β-values) were compared between schizophrenia and control subjects using ANOVA for all CpG sites. Three CpG sites, cg5 (*F* = 5.194, *p* = 0.028), cg7 (*F* = 5.753, *p* = 0.021) and cg10 (*F* = 6.155, *p* = 0.017) were hypermethylated in patients with schizophrenia compared to controls (Figure 3).

### 3.3. rs6314 and rs6313 Genotypes

Genotype frequencies were generated for schizophrenia and control subjects (Table 3). No T/T genotype was present in our samples for rs6314. For rs6314 we observed a significant difference between the genotype counts of schizophrenia and control subjects (*p* = 0.008) with an odds ratio (OR) of 5.7 (C/T as high risk genotype) while no association was detected between rs6313 and schizophrenia (*p* = 0.078). Although rs6313 association was not significant, individuals with the T/T genotype had an OR of 7.0 (*p* = 0.144). When analysed as a T-recessive trait, rs6313 was found associated with schizophrenia (*p* = 0.026) with an OR of 8.4 (T as high risk recessive allele). This suggests the T-allele is the schizophrenia risk allele for both rs6313 and rs6314 although results should be interpreted with caution due to the low sample number.

### 3.4. HTR2A mRNA Expression and Genotypes

ANOVA was performed to test the association between *HTR2A* mRNA expression and rs6314 and rs6313 genotypes. We compared the *HTR2A* mRNA expression between C/C genotypes and C/T genotypes for all samples, i.e., schizophrenia and control samples were pooled (there were no T/T genotypes present in our sample for rs6314). No differential expression was observed for either rs6314 (*F* = 0.160, *p* = 0.691) or rs6313 (*F* = 1.690, *p* = 0.198) genotypes.

### 3.5. DNA Methylation and Genotypes

For rs6314, ANOVA detected differential methylation between the subjects with C/T genotype and C/C genotype for cg3 (hypermethylated C/T group, *F* = 8.645, *p* = 0.005) and cg8 (hypermethylated C/T group, *F* = 5.800, *p* = 0.021). For rs6313, ANOVA detected differential methylation between C-allele carriers and subjects with the T/T genotype for cg6 (hypomethylated C-allele carriers, *F* = 4.184, *p* = 0.048).

### 3.6. Correlation between mRNA Expression and DNA Methylation

Using linear regression, we firstly tested the correlation between *HTR2A* mRNA expression and single site DNA methylation for the 10 CpG sites. No significant relationship was detected (Table S2). When all 10 CpG sites were analysed together, the combined effect of ten CpG sites on mRNA expression were not significant (R^2^ = 0.243, *p* = 0.291) although two CpG sites within the 10-CpG site model, cg2 (B = 0.282, *p* = 0.045) and cg4 (B = −0.328, *p* = 0.037) were significant.

We also tested for a correlation between *HTR2A* mRNA expression and single CpG site methylation for all 10 CpG sites within patients with schizophrenia as well as control subjects (Supplementary Table S2). For patients with schizophrenia, a significant correlation was identified for cg5 (B = 0.525, R^2^ = 0.196, *p* = 0.014) for single CpG site analysis. When all 10 CpG sites were analysed together in patients with schizophrenia, cg5 (B = 0.615, *p* = 0.007), cg6 (B = −0.695, *p* = 0.034), cg7 (B = 1.066, *p* = 0.026) and cg9 (B = −0.745, *p* = 0.049) were significantly correlated with mRNA expression with an overall R^2^ = 0.537 and overall significance of *p* = 0.008.

For control subjects, a significant correlation was identified for cg9 (B = 0.666, R^2^ = 0.272, *p* = 0.015) for single CpG site analysis. When all 10 CpG sites were analysed together in control subjects, no significantly correlation with mRNA expression was detected.

## 4. Discussion

This paper reports a number of complex associations with schizophrenia for the *HTR2A* gene that are potentially all interacting with each other. We found association between (1) schizophrenia and reduced *HTR2A* mRNA expression; (2) schizophrenia and hypermethylation of *HTR2A* promoter CpG sites (cg5, cg7 and cg10); and (3) schizophrenia and *HTR2A* genotypes for rs6314 and rs6313. Association was also found between DNA methylation and genotypes of both rs6314 and rs6313. There was a strong correlation between DNA methylation and mRNA expression in patients with schizophrenia.

No association was found between rs6314 and mRNA expression. This is consistent with Blasi et al. [21], who only found a trend of reduced *HTR2A* mRNA in prefrontal cortex samples bearing the T-allele of rs6314, but the trend was not significant (*p* = 0.06). Furthermore, they did not examine the effects of DNA methylation. The authors suggested, however, that rs6314 could alter splicing patterns with possible effects on *HTR2A* expression.

The polymorphism rs6314 was associated with differential methylation at CpG sites cg3 and cg8, though it is unclear what mechanism might lead to this considering that rs6314 is located 61 kb from the CpG sites in the promoter-exon I region. This is also consistent with Guhathakurta et al., who showed that rs6314 is unlikely to influence DNA methylation activity in the promoter (such as methylation site at rs6311) due to low LD [47]. Association was also detected between rs6313 and differential methylation of cg6. The rs6313 polymorphism is only 1.2 kb from cg6, so it is possible that it has a proximal effect on the binding of DNA methylases; further studies investigating the biological function of rs6313 on DNA methylation would clarify the role of rs6313 in schizophrenia.

An interesting observation in the mRNA expression–DNA methylation correlation analysis is the significant combined effect of all CpG sites detected when only patients with schizophrenia were analysed. Furthermore, methylation of cg5 and cg9 was correlated with mRNA expression and associated with schizophrenia. No significant combined effect of all CpG sites was detected when control-only subjects or pooled subjects (schizophrenia plus control) were analysed. This suggests that DNA methylation may have an effect on mRNA expression in patients with schizophrenia but not in control subjects. Perhaps there is little to no significant variation of mRNA expression and DNA methylation levels between control subjects for a significant correlation to be detected. However, it is possible that the significant correlation of DNA methylation-mRNA expression detected in schizophrenia may be a net result of DNA methylation alterations, along with other interacting factors (including genes, environmental factors and other underlying biological factors). Indeed, Abdolmaleky et al. found a negative correlation between HTR2A and COMT expression, a positive correlation between HTR2A and RELN expression, and a negative correlation between HTR2A expression and RELN promoter DNA methylation [10]. Overall, our results are consistent with those of Abdolmaleky et al., who reported an inverse relationship between *HTR2A* mRNA expression and promoter region DNA methylation. 

Our results showed an association between three hypermethylated CpG sites and schizophrenia. This is consistent with other studies that found hypermethylated DNA within the promoter region of *HTR2A* in schizophrenia brains and saliva [10,36], hypermethylated DNA at rs6313 in peripheral leukocytes in major psychosis patients with suicidal tendency [37] and hypermethylated *HTR2A* promoter CpG islands in the blood of patients with borderline personality disorder [48]. It should be noted that in a study by Abdolmaleky et al. that detected reduced *HTR2A* mRNA expression in brain samples, regions within the promoter were hypermethylated while regions near rs6313 within exon I were hypomethylated. To some extent, this observation is consistent with our findings, where both positive and negative correlations of DNA methylation-RNA expression are present. This suggests that the mechanisms regulating mRNA expression in brain are different in the blood and the saliva. Furthermore, combined CpG-site analysis suggests interaction between multiple CpG-sites resulting in altered *HTR2A* mRNA expression.

A limitation to this study is that, after correcting relevant results for multiple testing, they did not survive their respective thresholds. However, the samples are not completely independent so correction for multiple testing may not be valid.

This study has concentrated on DNA methylation in the promoter and exon I regions of *HTR2A*. Future studies should explore the −3′ region of the gene to investigate methylation alteration in schizophrenia. These studies should be conducted in relevant brain tissues as it is the organ of major pathology for schizophrenia. In addition to age and PMI, factors influencing the outcomes of mRNA expression and DNA methylation including stress, medication and substance use, nutrition history, comorbidities, ethnogeographic information and other environmental factors should be included in future studies to further improve the accuracy of the findings.

## 5. Conclusions

In conclusion, we report that there is an association between schizophrenia and three separate factors: reduced *HTR2A* mRNA expression, hypermethylation of *HTR2A* promoter CpG sites (cg5, cg7 and cg10) and genetic association with *HTR2A* genotypes for rs6314 and rs6313. The association of the T-allele of rs6314 and rs6313 with schizophrenia may be due to an alteration in biological mechanisms other than mRNA expression regulation. The reduced expression of *HTR2A* mRNA in schizophrenia patients may be mainly attributed to DNA methylation of CpG sites within the promoter-exon I region, and possibly influenced by other underlying factors. Therefore, epigenetic *HTR2A* regulation may affect brain function, which contributes to the development of schizophrenia.

## Figures and Tables

**Table 1:** Summary of demographic D=details.

	Control (*n* = 23)	Schizophrenia (*n* = 22)	*t*-Test/Chi-Square *p*-Value
Age, mean (s.d.)	70.2 (9.2)	52.5 (22.7)	0.0022
PMI, mean (s.d.)	14.1 (3.2)	24 (10.6)	0.0003
Sex, male (%)	18 (78)	14 (64)	0.2778

s.d., one standard deviation.

**Table 2:** Details of selected CpG sites.

CpG Site	Illumina CpG Site Name	Feature	Locus
cg1	cg20102280	Exon I	chr13:46896658
cg2	cg15894389	Exon I	chr13:46896722
cg3	cg02250787	Exon I	chr13:46896854
cg4	cg06476131	Exon I	chr13:46896917
cg5	cg16188532	Exon I	chr13:46896955
cg6	cg09361691	Exon I	chr13:46897034
cg7	cg11514288	Exon I	chr13:46897062
cg8	cg27068143	Promoter	chr13:46897129
cg9	cg10323433	Promoter	chr13:46897427
cg10	cg02027079	Promoter	chr13:46897570

**Table 3:** Genotype and allele frequencies for rs6314 in schizophrenia and control groups.

	Genotype/Allele	Schizophrenia	Control	Chi-Square *p*-Value	Odds Ratio
rs6314 Genotype	C/C	10	19	0.008	5.7
	C/T	12	4		
	T/T	0	0		
	Total	22	23		
rs6314 Allele	C	32	42	0.019	3.9
	T	12	4		
	Total	44	46		
rs6313 Genotype	C/C	6	7	0.078	--
	C/T	9	14		
	T/T	6	1		
	Total	21	22		
rs6313 Allele	C	21	28	0.201	--
	T	21	16		
	Total	42	44		

## Supplementary Materials

The following are available online at www.mdpi.com/2073-4425/8/1/14/s1, Supplementary Table S1: Complete information of sample age, post-mortem interval (PMI) and gender; Supplementary Table S2: Linear regression results (age and PMI adjusted) of HTR2A mRNA expression and DNA methylation for 10 CpG sites for (a) all subjects (schizophrenic plus control); (b) schizophrenic subjects; and (c) control subjects.
