# Supplementary material for: mRNA Expression and DNA Methylation Analysis of Serotonin Receptor 2A (HTR2A) in the Human Schizophrenic Brain
Source: Genes (Basel). 2017 Jan 4;8(1):14. doi: 10.3390/genes8010014 (PMC5295009; doi:10.3390/genes8010014)
Supplement: Supplementary file 1 [file genes-08-00014-s001.zip › genes-153986-suppl.-final/genes-153986-supplementary-english.docx]

Supplementary Materials: mRNA Expression and DNA Methylation Analysis of Serotonin Receptor 2A (*HTR2A*) in the Human Schizophrenic Brain

Sern-Yih Cheah, Bruce R. Lawford, Ross McD. Young, Charles P. Morris and Joanne Voisey

**Table S2.** Linear regression results (age and PMI adjusted) of *HTR2A* mRNA expression and DNA methylation for 10 CpG sites for (**a**) all subjects (schizophrenic plus control); (**b**) schizophrenic subjects; and (**c**) control subjects.

| (**a**) |  |  |  |
| --- | --- | --- | --- |
| **CpG Site** | **Coefficient** | **R^2^** | ***P*** |
| cg1 | 0.119 | 0.014 | 0.443 |
| cg2 | 0.131 | 0.029 | 0.262 |
| cg3 | −0.026 | 0.000353 | 0.903 |
| cg4 | −0.024 | 0.074 | 0.069 |
| cg5 | −0.045 | 0.003 | 0.705 |
| cg6 | −0.017 | 0.000265 | 0.916 |
| cg7 | 0.042 | 0.002 | 0.782 |
| cg8 | 0.009 | 0.000031 | 0.971 |
| cg9 | 0.069 | 0.003 | 0.706 |
| cg10 | −0.122 | 0.009 | 0.538 |
| (**b**) |  |  |  |
| **CpG Site** | **Coefficient** | **R^2^** | ***P*** |
| cg1 | 0.283 | 0.023 | 0.435 |
| cg2 | 0.091 | 0.011 | 0.589 |
| cg3 | −0.119 | 0.007 | 0.679 |
| cg4 | −0.213 | 0.046 | 0.267 |
| cg5 | 0.525 | 0.196 | 0.014 |
| cg6 | −0.111 | 0.010 | 0.607 |
| cg7 | 0.080 | 0.005 | 0.722 |
| cg8 | −0.262 | 0.018 | 0.494 |
| cg9 | −0.114 | 0.010 | 0.608 |
| cg10 | −0.230 | 0.029 | 0.384 |
| (**c**) |  |  |  |
| **CpG Site** | **Coefficient** | **R^2^** | ***P*** |
| cg1 | 0.138 | 0.040 | 0.387 |
| cg2 | 0.138 | 0.040 | 0.386 |
| cg3 | 0.183 | 0.021 | 0.535 |
| cg4 | −0.210 | 0.074 | 0.232 |
| cg5 | −0.114 | 0.036 | 0.410 |
| cg6 | 0.436 | 0.154 | 0.077 |
| cg7 | 0.265 | 0.087 | 0.194 |
| cg8 | 0.381 | 0.066 | 0.258 |
| cg9 | 0.666 | 0.272 | 0.015 |
| cg10 | 0.472 | 0.127 | 0.111 |
